# Promoter methylation of PTEN gene in high grade gliomas

**DOI:** 10.1186/1755-8166-7-S1-P10

**Published:** 2014-01-21

**Authors:** C Lavanya, GK Chetan, MK Sibin, Manoj M Jeru, Bharath MM Srinivas

**Affiliations:** 1 Department of Human genetics National Institute of Mental Health and Neurosciences, Bangalore, India; 2 Department of Neurochemistry, National Institute of Mental Health and Neurosciences, Bangalore, India

## Background

Gliomas are the most common primary malignant central nervous system tumors arising from glial cells accounting for 80% of all malignant brain tumors. Many mutations occur frequently in genes that control cell cycle and proliferation leading to tumor progression. Major genes that are associated with the malignant tumors are MGMT, hTERT, TP53, PTEN, P16. Phosphatase and tensin homologue deleted on chromosome 10 (PTEN) is a tumor suppressor gene deleted or mutated in many cancers. It plays a significant role in cellular processes like cell cycle progression, apoptosis, translation, growth, proliferation and migration by negatively controlling PI3/AKt pathway. Deregulation of PI3K signaling pathways proceed from genetic alterations in the PTEN gene on 10q23 at the level of mutation, LOH and methylation identified in 60% of glioblastoma. Recent reports show that absence of PTEN was associated with increased tumor malignancy in gliomas. This seems to have prognostic significance and accepted as independent prognostic factor. In this study 15 high grade glioma tissues were collected from neurosurgery department and confirmed as glioma histologically.

## Material and methods

DNA was isolated using Qiagen kit and bisulphite DNA conversion was done by Qiagen Epitect. PCR was performed to check the methylation status in the promoter region of PTEN gene.

## Results

We noticed that around 50% of patients DNA show methylation in promoter region of PTEN gene when compared with healthy control DNA. We are looking at the response of these patients to chemotherapy and radiotherapy. We are also looking in to changes in the PI3/AKt signaling pathway.

## Conclusions

There is a possibility of correlation between the methylation status and patients survival.

